# An Important Decision

**Published:** 1898-10

**Authors:** 


### An Important Decision.

A decision of unusual interest to the medical profession throughout the world has lately been handed down by the United States Supreme Court. In 1878 Dr. Benj. W. Hawker, a legally qualified practitioner of the State of New York, was convicted of a felony, viz.: performing a criminal abortion; and was sentenced to imprisonment for ten years. At the expiration of his time of servitude he attempted to resume practice, with the result that the Medical Society of the County of New York brought suit against him for violation of a State law. His counsel argued that a construction of the law making it illegal to practice medicine after conviction of a felony is unjust and unconstitutional, inasmuch as it in effect adds a new punishment for the crime. The people contended, however, that the State has the right to exact good moral character as one of the qualifications for the practice of medicine. The first trial resulted in a verdict of guilty and the imposition of a fine; the case was appealed and the judges of the Appellate Court decided to set aside the conviction, one judge (Ingraham) delivering a vigorous dissenting opinion. On a final appeal to the United States Supreme Court, nine judges confirmed the conviction and sustained the constitutionality of the law, citing many decisions in support of their position. This decision will, therefore, stand as law for all future time, and will debar any man or woman convicted of a felony from practicing medicine.—*American Journal of Surgery and Hygiene.*

